# Complete genomes of diverse *Escherichia coli* isolates lacking canonical virulence factors from pediatric sources

**DOI:** 10.1128/mra.01128-25

**Published:** 2026-03-04

**Authors:** David A. Rasko, Bernadette A. Hritzo, Jane M. Michalski Wilhelm

**Affiliations:** 1 Institute for Genome Sciences, University of Maryland School of Medicine 12264, Baltimore, Maryland, USA; 2 Department of Microbiology and Immunology, University of Maryland School of Medicine 12264, Baltimore, Maryland, USA; 3 Center for Pathogen Research, University of Maryland School of Medicine 12264, Baltimore, Maryland, USA; 4 Department of Microbial Pathogenesis, University of Maryland School of Dentistry 89015, Baltimore, Maryland, USA; University of Pittsburgh School of Medicine, Pittsburgh, Pennsylvania, USA

**Keywords:** *Escherichia coli*, genome, commensal

## Abstract

Knowledge of the genomic diversity of *Escherichia coli* is largely derived from the pathogenic isolates associated with human or animal infection. We describe the complete genomes of *E. coli* isolates lacking canonical virulence gene markers from children enrolled in the Global Enteric Multicenter Study.

## ANNOUNCEMENT

The majority of knowledge regarding the genomic diversity of *Escherichia coli* is derived from the analysis of pathogenic isolates associated with human or animal infection. In this announcement, we describe the genomes of 22 diverse *E. coli* isolates lacking canonical virulence genes isolated from subjects enrolled in the Global Enteric Multicenter Study (1, 2).

Initial isolation was conducted as described by Panchalingam et al. (3). Briefly, feces or rectal swabs were transported in cold containers and inoculated into transport media and then selective growth media for multiple pathogens within 6 or 18 h of collection. Several lactose-fermenting colonies were selected from McConkey agar (incubated for 48 h at 37°C) and then sub-cultured in the motility indole ornithine medium. Suspected *E. coli* isolates (lactose [+], indole [−], methyl red [+], Voges-Proskauer [−], and citrate [−]) were categorized into pathogenic types by multiplex PCR (4). Multiplex PCR examined the following canonical virulence genes: enterotoxigenic *E. coli* heat-labile (*elt*) and heat-stable enterotoxin (*est*), enteropathogenic *E. coli* (EPEC) intimin (*eae*), EPEC plasmid-encoded bundle-forming pilus (*bfpA*), enteroaggregative *E. coli* (EAEC) plasmid-encoded *aatA*, and the EAEC chromosomally encoded *aaiC* (3, 4). Verified *E. coli* isolates were stored at 80°C in 20% glycerol at the Center for Vaccine Development at the University of Maryland School of Medicine.

Genomically and phylogenomically diverse isolates were selected for complete genome sequencing from diarrheal case and control samples, as well as from male and female subjects, lacking a PCR amplicon for assayed virulence genes. Isolates were resurrected from frozen culture on Lysogeny agar overnight at 37°C. Genomic DNA was extracted using Qiagen’s DNeasy Blood & Tissue Kit, and sequencing libraries for both sequencing technologies were generated with unsheared genomic DNA. Nanopore libraries were prepared using Oxford Nanopore’s “Genomic DNA by Ligation” kit (5) and run on Nanopore R9 flow cells with the MinION Mk1B sequencer. Basecalling was performed using Guppy (v4.2.2) (6). Illumina libraries (150 bp paired-end) were prepared using the Kapa Library Kit (Roche/Illumina) and sequenced on the Illumina HiSeq 4000 platform. Quality control and adapter trimming were performed with bcl2fastq (v2.20.0.445) (7) and porechop (v0.2.3_seqan2.1.1) (8) for Illumina and ONT sequencing, respectively. Hybrid assembly using both Illumina and ONT reads was conducted, and completeness was assessed with Unicycler (v0.4.8) (9). Final assembly statistics were recorded with QUAST (5.0.2) (10). Genomes were annotated with the NCBI prokaryotic genome annotation pipeline (v6.10) (11). All software was run with default values.

Table 1 contains isolate information, sequencing statistics, and assembly GenBank accession numbers. Average genome coverage was 385.4× (range: 243–555×). The assembled complete genomes have an average genome size of 5,143,766 bp (range: 4,492,591–5,537,088 bp) and an average GC% of 50.63% (range: 50.3%–51.0%). Genomic analysis included determination of the sequence type (MLST with BIGSdb) (12, 13) and molecular serotype (Serotype Finder v. 1.1 [14, 15]). The 22 genomes include representative diarrheal case and control isolates from the seven GEMS geographic sites (Table 1). These isolates represent 7 phylogenomic groups (2), 16 sequence types, and 18 serotypes (2, 14, 15). These data provide detailed information on the genomic and antigenic diversity of keystone members of the microbiome.

**TABLE 1:** Isolate and genome metrics

Isolate Information								Sequencing Statistics											Accession Numbers			
Isolate	Country	Case/ Control	Sex	Year of isolation	Phylogenetic Group	Serotype	ST (CC)^*a*^	Molecule name	Size (bp)	Illumina Read Pairs	Illumina Bases	ONT Trimmed Reads	ONT Trimmed Bases	ONT N50	Genome Coverage	# contigs	Total genome length (bp)	GC (%)	BioProject	Assembly	SRA - Illumina	SRA - ONT
100117	Gambia	Case	Female	2008	cryptic	OND:H45	485	E100117	5,053,006	3,198,198	983,510,344	157,308	513,659,526	3,299.50	273.3	6	5,478,516	50.25	PRJNA1321367	JBRERF010000001	SRR35329244	SRR35329229
								pE100117_127	127,487											JBRERF010000002		
								pE100117_123	123,295											JBRERF010000003		
								pE100117_121	121,255											JBRERF010000004		
								pE100117_50	50,722											JBRERF010000005		
								pE100117_3	2751											JBRERF010000006		
100181	Gambia	Control	Female	2008	E	O138:H28	2614	E100181	4,796,279	3,503,198	1,080,153,114	299,024	1,094,185,570	3,677.90	439.6	2	4,946,088	50.74	PRJNA1321367	JBRERE010000001	SRR35329243	SRR35329228
								pE100181_149	149,809											JBRERE010000002		
100248	Gambia	Control	Female	2008	cryptic	OND:H45	485	E100248	5,051,491	3,185,545	980,331,714	395,689	984,764,733	2,514.20	354.9	7	5,537,088	50.28	PRJNA1321367	JBRERD010000001	SRR35329232	SRR35329227
								pE100248_127	127,487											JBRERD010000002		
								pE100248_123	123,295											JBRERD010000003		
								pE100248_121	121,244											JBRERD010000004		
								pE100248_60	60,098											JBRERD010000005		
								pE100248_50	50,722											JBRERD010000006		
								pE100248_3	2751											JBRERD010000007		
100416	Gambia	Case	Female	2008	E	O52:H45	ND^*b*^	E100416	5,080,683	3,606,837	1,109,416,821	677,108	1,731,711,741	2,572.90	548.5	7	5,179,586	50.64	PRJNA1321367	JBRERC010000001	SRR35329221	SRR35329226
								pE100416_79	79,074											JBRERC010000002		
								pE100416_7	6768											JBRERC010000003		
								pE100416_6	6721											JBRERC010000004		
								pE100416_3	2690											JBRERC010000005		
								pE100416_2	2101											JBRERC010000006		
								pE100416_1	1549											JBRERC010000007		
200088	Mali	Control	Female	2008	A	O9:H9	10 (10)	E200088	4,704,959	3,524,483	1,085,557,797	288,802	529,597,662	1,862	343.3	1	4,704,959	50.9	PRJNA1321367	JBRERB010000001	SRR35329210	SRR35329225
200112	Mali	Case	Male	2008	A	O51:H5	43 (10)	E200112	4,785,677	3,714,584	1,139,501,669	518,572	1,152,766,893	2,252.80	475.0	2	4,825,512	50.63	PRJNA1321367	JBRERA010000001	SRR35329205	SRR35329224
								pE200112_40	39,835											JBRERA010000002		
200164	Mali	Control	Female	2008	G	O8:H16	1163	E200164	4,873,132	3,471,705	1,069,971,171	1,845,803	1,350,690,608	782.9	480.8	5	5,035,080	50.9	PRJNA1321367	JBREQZ010000001	SRR35329204	SRR35329223
								pE200164_150	150,299											JBREQZ010000002		
								pE200164_5	4772											JBREQZ010000003		
								pE200164_4	4064											JBREQZ010000004		
								pE200164_3	2813											JBREQZ010000005		
200177	Mali	Case	Male	2008	G	O143:H4	117	E200177	4,965,812	3,529,673	1,090,533,549	1,199,958	1,357,232,844	1,173.40	466.9	8	5,242,659	50.61	PRJNA1321367	JBREQY010000001	SRR35329203	SRR35329222
								pE200177_185	185,318											JBREQY010000002		
								pE200177_59	59,223											JBREQY010000003		
								pE200177_8	8472											JBREQY010000004		
								pE200177_7	6754											JBREQY010000005		
								pE200177_6	6673											JBREQY010000006		
								pE200177_5	5274											JBREQY010000007		
								pE200177_5_1	5133											JBREQY010000008		
300033	Mozambique	Case	Female	2008	D	O17/O77:H18	449 (31)	E300033	5,080,988	3,530,865	1,086,765,395	641,206	1,258,426,676	1,986.20	461.6	1	5,080,988	50.57	PRJNA1321367	JBREQX010000001	SRR35329202	SRR35329220
300081	Mozambique	Case	Female	2008	A	O9:H4	1421	E300081	4,714,667	3,503,147	1,063,510,989	209,141	1,548,586,555	7,450.70	525.5	4	4,970,282	50.63	PRJNA1321367	JBREQW010000001	SRR35329201	SRR35329219
								pE300081_246	246,797											JBREQW010000002		
								pE300081_7	6790											JBREQW010000003		
								pE300081_2	2028											JBREQW010000004		
300444	Mozambique	Control	Female	2008	D	O125ab:H21	ND^*b*^	E300444	5,030,166	3,394,232	1,043,614,492	138,477	343,308,685	2,492.70	262.2	3	5,289,403	50.55	PRJNA1321367	JBREQV010000001	SRR35329242	SRR35329218
								pE300444_144	144,152											JBREQV010000002		
								pE300444_115	115,085											JBREQV010000003		
300490	Mozambique	Control	Male	2008	A	O21:H52	ND^*b*^	E300490	4,592,591	4,038,317	1,243,136,201	62,567	207,607,188	3,357.40	315.9	1	4,592,591	50.69	PRJNA1321367	JBREQU010000001	SRR35329241	SRR35329217
400625	Kenya	Control	Female	2008	B2	O6:H1	73 (73)	E400625	5,247,215	4,592,832	1,415,622,561	360,598	564,594,576	1,598.20	377.4	1	5,247,215	50.41	PRJNA1321367	JBREQT010000001	SRR35329240	SRR35329216
400660	Kenya	Case	Male	2008	B1	OND:H10	155 (155)	E400660	4,886,029	3,592,460	1,108,305,408	1,542,713	1,652,950,513	1,115.60	554.6	3	4,979,024	50.77	PRJNA1321367	JBREQS010000001	SRR35329239	SRR35329215
								pE400660_84	84,026											JBREQS010000002		
								pE400660_9	8969											JBREQS010000003		
401651	Kenya	Control	Male	2008	B1	OND:H12	2522	E401651	5,055,475	2,863,107	883,519,435	296,760	429,591,983	1,477	242.6	7	5,412,210	50.67	PRJNA1321367	JBREQR010000001	SRR35329238	SRR35329214
								pE401651_221	221,507											JBREQR010000002		
								pE401651_67	67,429											JBREQR010000003		
								pE401651_55	55,829											JBREQR010000004		
								pE401651_7	6790											JBREQR010000005		
								pE401651_3	2707											JBREQR010000006		
								pE401651_2	2473											JBREQR010000007		
401656	Kenya	Case	Male	2008	B2	O6:H1	73 (73)	E401656	5,280,818	3,320,103	1,020,837,383	252,612	468,187,930	1,883.40	274.8	3	5,417,921	50.43	PRJNA1321367	JBREQQ010000001	SRR35329237	SRR35329213
								pE401656_135	135,557											JBREQQ010000002		
								pE401656_2	1546											JBREQQ010000003		
500010	India	Case	Male	2007	F	O86:H30	648 (648)	E500010	5,189,890	3,312,203	1,019,046,898	192,306	896,415,161	4,681.20	359.2	5	5,331,963	50.58	PRJNA1321367	JBREQP010000001	SRR35329236	SRR35329212
								pE500010_130	130,329											JBREQP010000002		
								pE500010_6	6222											JBREQP010000003		
								pE500010_4	4063											JBREQP010000004		
								pE500010_2	1459											JBREQP010000005		
500111	India	Control	Female	2007	F	O1:H6	648 (648)	E500111	5,276,337	3,209,339	988,004,218	716,379	656,220,183	952.4	302.7	5	5,431,682	50.54	PRJNA1321367	JBREQO010000001	SRR35329235	SRR35329211
								pE500111_146	146,567											JBREQO010000002		
								pE500111_4	4063											JBREQO010000003		
								pE500111_3	3256											JBREQO010000004		
								pE500111_2	1459											JBREQO010000005		
600029	Bangladesh	Control	Male	2007	A	OND:H20	1602	E600029	4,755,045	3,536,274	1,087,109,317	560,627	710,610,778	1,296.70	359.5	6	5,000,542	50.94	PRJNA1321367	JBREQN010000001	SRR35329234	SRR35329209
								pE600029_86	86,282											JBREQN010000002		
								pE600029_80	80,796											JBREQN010000003		
								pE600029_66	65,902											JBREQN010000004		
								pE600029_8	8419											JBREQN010000005		
								pE600029_4	4098											JBREQN010000006		
600055	Bangladesh	Case	Male	2007	A	OND:H20	8308	E600055	4,596,415	3,671,249	1,134,729,601	293,089	398,740,472	1,384.80	315.3	10	4,863,499	50.98	PRJNA1321367	JBREQM010000001	SRR35329233	SRR35329208
								pE600055_103	103,563											JBREQM010000002		
								pE600055_76	76,328											JBREQM010000003		
								pE600055_36	36,260											JBREQM010000004		
								pE600055_15	15,542											JBREQM010000005		
								pE600055_11	11,167											JBREQM010000006		
								pE600055_10	10,215											JBREQM010000007		
								pE600055_7	6755											JBREQM010000008		
								pE600055_4	4082											JBREQM010000009		
								pE600055_3	3172											JBREQM010000010		
700462	Pakistan	Control	Male	2008	C	OND:H5	977	E700462	5,160,326	3,335,822	1,022,957,949	658,675	860,307,408	1,341.80	355.0	4	5,304,698	50.52	PRJNA1321367	JBREQL010000001	SRR35329231	SRR35329207
								pE700462_93	92,979											JBREQL010000002		
								pE700462_45	45,193											JBREQL010000003		
								pE700462_6	6200											JBREQL010000004		
700481	Pakistan	Case	Male	2008	C	OND:H12	ND^*b*^	E700481	4,941,301	3,617,689	1,114,369,729	636,158	948,404,354	1,505.20	389.8	9	5,291,352	50.63	PRJNA1321367	JBREQK010000001	SRR35329230	SRR35329206
								pE700481_123	122,863											JBREQK010000002		
								pE700481_122	121,613											JBREQK010000003		
								pE700481_91	91,137											JBREQK010000004		
								pE700481_5	5813											JBREQK010000005		
								pE700481_3	3272											JBREQK010000006		
								pE700481_2.1	2101											JBREQK010000007		
								pE700481_2	2012											JBREQK010000008		
								pE700481_1.2	1240											JBREQK010000009		

^*a*^ Numbers in parentheses indicate clonal complexes for *E. coli*.

^*b*^ Not determined; includes single and/or double locus variants as well as novel STs.

## Data Availability

All data have been released under BioProject PRJNA1321367. The associated SRA and genome assembly accession numbers for each isolate are listed in Table 1.
